# Molecular basis and functional consequences of the interaction between the base excision repair DNA glycosylase NEIL1 and RPA

**DOI:** 10.1016/j.jbc.2024.107579

**Published:** 2024-07-25

**Authors:** Rémy A. Le Meur, Turner J. Pecen, Kateryna V. Le Meur, Zachary D. Nagel, Walter J. Chazin

**Affiliations:** 1 Departments of Biochemistry and Chemistry, Center for Structural Biology, Vanderbilt University, Nashville, Tennessee, USA; 2 Department of Environmental Health, Harvard T.H. Chan School of Public Health, Boston, Massachusetts, USA

**Keywords:** DNA glycosylase, base excision repair, NMR spectroscopy, isothermal titration calorimetry, fluorescence Multiplex-Host cell Reactivation reporter assay, nucleotide excision repair

## Abstract

NEIL1 is a DNA glycosylase that recognizes and initiates base excision repair of oxidized bases. The ubiquitous ssDNA binding scaffolding protein, replication protein A (RPA), modulates NEIL1 activity in a manner that depends on DNA structure. Interaction between NEIL1 and RPA has been reported, but the molecular basis of this interaction has yet to be investigated. Using a combination of NMR spectroscopy and isothermal titration calorimetry (ITC), we show that NEIL1 interacts with RPA through two contact points. An interaction with the RPA32C protein recruitment domain was mapped to a motif in the common interaction domain (CID) of NEIL1 and a dissociation constant (Kd) of 200 nM was measured. A substantially weaker secondary interaction with the tandem RPA70AB ssDNA binding domains was also mapped to the CID. Together these two contact points reveal NEIL1 has a high overall affinity (Kd ∼ 20 nM) for RPA. A homology model of the complex of RPA32C with the NEIL1 RPA binding motif in the CID was generated and used to design a set of mutations in NEIL1 to disrupt the interaction, which was confirmed by ITC. The mutant NEIL1 remains catalytically active against a thymine glycol lesion in duplex DNA *in vitro*. Testing the functional effect of disrupting the NEIL1-RPA interaction *in vivo* using a Fluorescence Multiplex-Host Cell Reactivation (FM-HCR) reporter assay revealed an unexpected role for NEIL1 in nucleotide excision repair. These findings are discussed in the context of the role of NEIL1 in replication-associated repair.

Reactive Oxygen Species (ROS) generated by endogenous and exogenous agents provide a constant source of damage to DNA (1). If left unrepaired, oxidation of bases caused by ROS can alter the base-pairing of DNA. Mispairing, in turn, can lead to fork instability and the generation of mutations that can ultimately cause a variety of diseases including cancer, expedited aging, and neurodegeneration (2, 3). Single-strand DNA (ssDNA), which exists transiently during replication, transcription, and recombination, is highly sensitive to ROS. The most common reactions are cytosine deamination into uracil, alkylation of adenine and cytosine, and spontaneous depurination and depyrimidination (1, 4, 5, 6). These and other oxidized bases are primarily repaired by the Base Excision Repair (BER) pathway. Although repair of oxidized bases is essential for genome stability, incomplete repair in the context of replication can lead to highly toxic strand breaks (7). Therefore, tight coordination between BER and the replication machinery is essential for genome maintenance in replicating cells.

NEIL1 is a DNA glycosylase that initiates BER of oxidized bases and is critical for pre-replicative DNA repair (8). Among the 5 mammalian DNA glycosylases identified to initiate BER of an oxidized base (OGG1, NTH1, NEIL1, NEIL2, and NEIL3), only NEIL1 and NEIL2 are effective on both double-stranded (ds) DNA and ssDNA (9, 10). NEIL1 expression is up-regulated in S-phase and interacts not only with BER enzymes, such as PolB, Lig3, and XRCC1 but also with replication enzymes such as PCNA and RPA (11). PCNA stimulates NEIL1 excision of damaged bases. This interaction suggests a role for NEIL1 in the surveillance of DNA as the replication fork progresses (11). RPA stimulates NEIL1 activity when the damage is present in dsDNA near a ssDNA junction, as in pre-replicative DNA. Conversely, RPA inhibits the excision of a damaged base by NEIL1 in ssDNA (12). This function is believed to help prevent the formation of toxic strand breaks. Thus, there is substantial evidence that the interaction between RPA and NEIL1 is crucial to the regulation of BER during replication; however, a detailed molecular understanding of this interaction and its consequences is not yet available.

NEIL1 has a primary catalytic domain and a 100-residue disordered C-terminal extension that mediates interactions with its partner proteins including RPA. RPA, the ubiquitous ssDNA binding protein in eukaryotes, is a trimer comprised of RPA70, RPA32, and RPA14 sub-units. The interaction between RPA and NEIL1 has been previously characterized using a combination of deletion mutagenesis, co-immunoprecipitation, in-vitro pull-down, far Western, and fluorescence binding assays (12). Binding to RPA was coarsely mapped to the RPA70 sub-unit and was shown to involve NEIL1 residues 289 to 349 within the C-terminal disordered extension termed the Common Interaction Domain (CID). Here we report a detailed study of the molecular basis of NEIL1-RPA interaction using a combination of NMR, isothermal titration calorimetry (ITC), computational and cell-based Fluorescence Multiplex-Host Cell Reactivation (FM-HCR) reporter assays. The design, generation, and validation of a specific NEIL1 mutant inhibiting the physical interaction between NEIL1 and RPA provided a valuable reagent to test the functional effects of suppressing this interaction. Leveraging the unique ability of the FM-HCR to simultaneously probe multiple DNA repair pathways, we discovered an unexpected role for NEIL1 in nucleotide excision repair (NER). These results suggest potential roles for NEIL1-RPA interaction in replication-associated base excision repair.

## Results

### RPA interaction with NEIL1 is mediated by RPA 32C and 70AB domains

**Figure 1:**
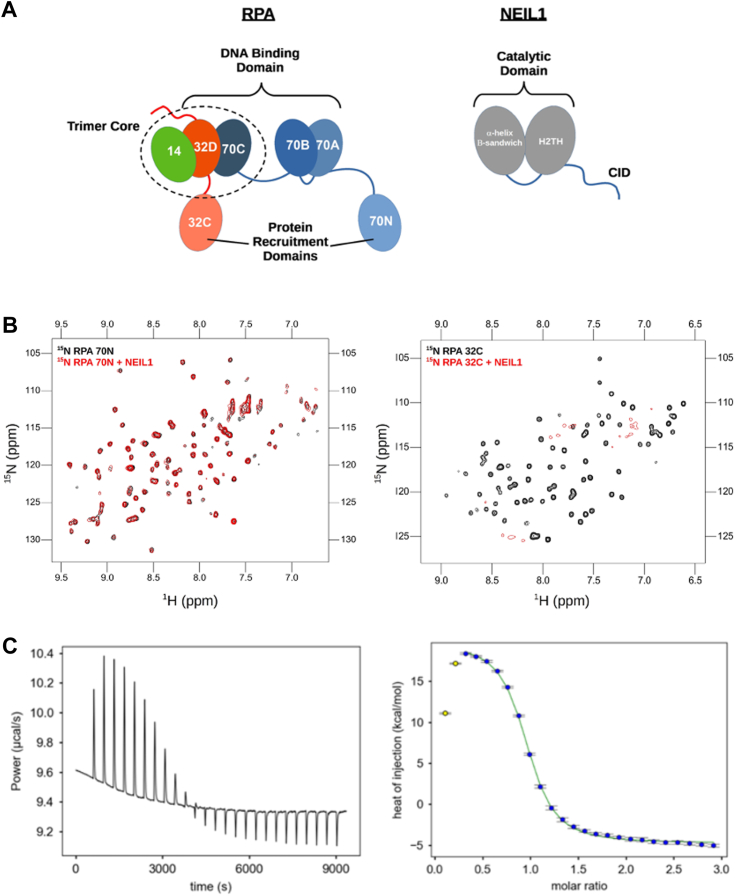
**Interaction of NEIL1 with the RPA70N and RPA32C protein recruitment domains**. *A*, schematic of RPA (*left*) and Neil1 (*right*) domain architecture. RPA is an heterotrimer of RPA70, RPA32 and RPA14 with 7 Oligonucleotide/Oligosaccharide-Binding (OB-fold) domains (70N, 70A, 70B, 70D, 32D, and 14) and a winged helix domain (32C). RPA 14, 32D and 70C form the trimer core. 70A, 70B, 70C, and 70D form the DNA binding apparatus. RPA 70N and 32C are protein recruitment domains. NEIL1 contains a catalytic domain with the typical structure of the Fpg/Nei superfamily: a first domain composed of an α-helix and β-sandwich tethered to a Helix two-turn helix (H2TH) domain. NEIL1 also contains an unstructured C-terminal domain harboring a Common Interaction Domain (CID), involved in protein-protein interactions. *B*, ^15^N-^1^H HSQC spectra recorded at 800 MHz of ^15^N-enriched RPA70N (*left*) and RPA32C (*right*) in the absence (*black*) or presence (*red*) of 4 M equivalents of NEIL1. Spectra were acquired at 25 °C in a buffer containing 25 mM Tris-HCl pH 7.5, 150 mM NaCl, and 1 mM DTT. *C*, isothermal titration calorimetry experiment for RPA32C titrated with NEIL1. Data were acquired at 25 °C in a buffer containing 50 mM HEPES pH 7.5, 150 mM NaCl, 10% glycerol, and 0.5 mM TCEP. A Kd value of 240 nM was extracted from the data.

The domain structures of NEIL1 and RPA are shown in Figure 1*A*. To identify which domains of RPA interact with NEIL1, two-dimensional ^15^N-^1^H Heteronuclear Single Quantum Coherence (HSQC) NMR experiments were acquired to monitor perturbations of backbone ^15^N and ^1^H chemical shifts (CSPs). Following the strategy of past studies characterizing RPA interaction partners, ^15^N-enriched samples were prepared for the two protein recruitment domains RPA 32C and 70N, and the tandem high affinity DNA binding domains RPA70AB. In these experiments, the binding of 44 kDa NEIL1 is expected to result in substantial line broadening of the signals from the RPA domain(s).

Previous studies mapped the binding of NEIL1 to the RPA70 subunit, which contains the RPA70N protein recruitment domain (12). However, upon the addition of up to 4 M equivalents of NEIL1, no significant perturbations of the spectrum of RPA70N were observed (Fig. 1*B*). In contrast, the addition of NEIL1 to ^15^N-enriched RPA 32C resulted in substantial line broadening throughout the whole spectrum (Fig. 1*B*), indicating a significant interaction. To obtain deeper insights, the binding of RPA32C by NEIL1 was characterized by Isothermal Titration Calorimetry (ITC) (Fig. 1*C*). These data revealed that binding was a primarily endothermic enthalpy-driven process with a dissociation constant (Kd) of 240 nM.

**Figure 2:**
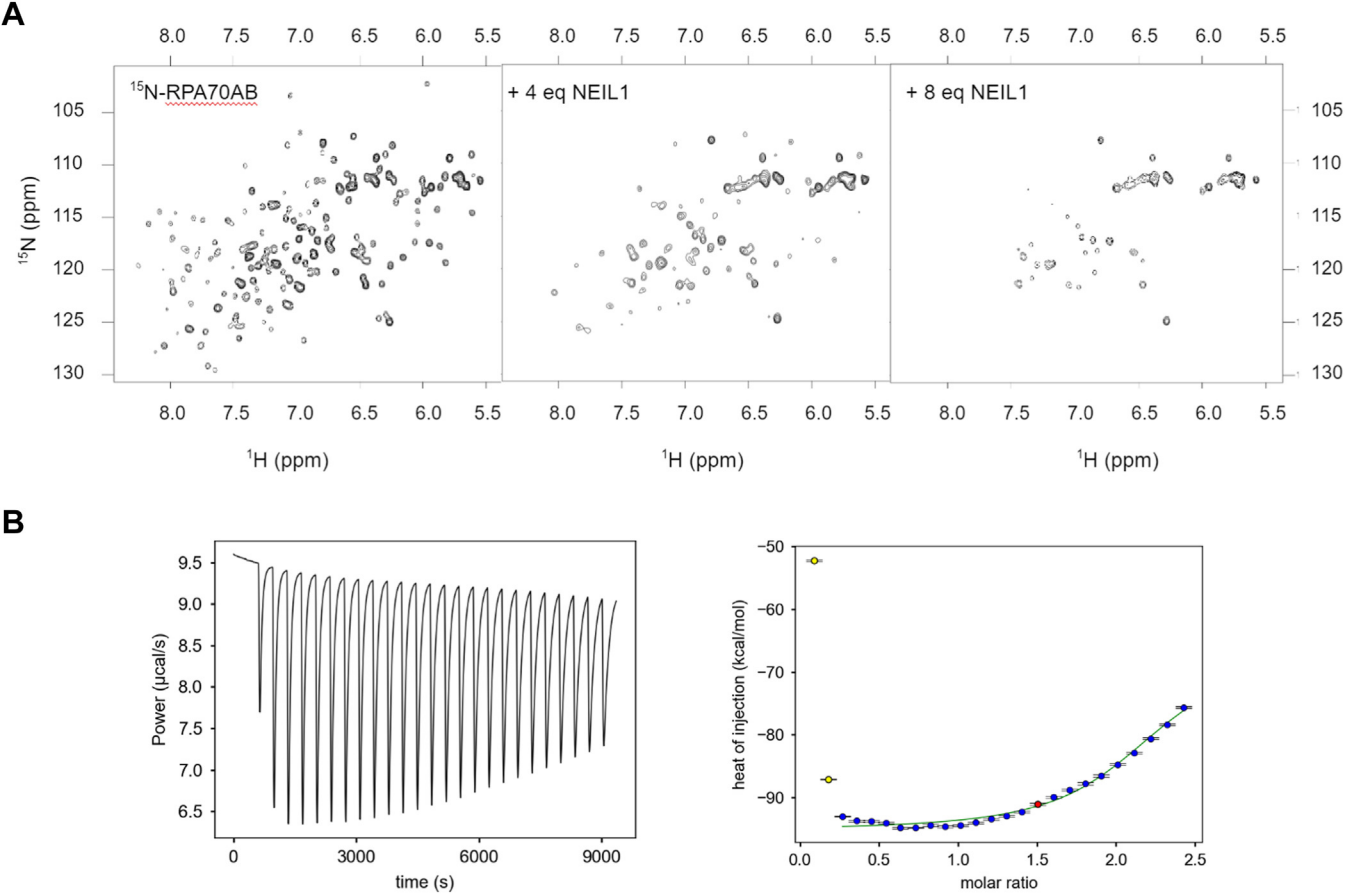
**Interaction of NEIL1 with the tandem high affinity DNA binding domains RPA70AB**. *A*, ^15^N-^1^H HSQC spectra recorded at 800 MHz of ^15^N-enriched RPA70AB free and in the presence of 4 and 8 equivalents of NEIL1. Spectra were acquired at 25 °C in a buffer containing 25 mM Tris/HCl pH 7.5, 150 mM NaCl, and 1 mM DTT. *B*, isothermal titration calorimetry experiment for RPA70AB titrated with NEIL1. Data were acquired at 25 °C in a buffer containing 50 mM HEPES pH7.5, 150 mM NaCl, 10% glycerol, and 0.5 mM TCEP. The binding of RPA70AB is so weak that it is not possible to quantify the thermodynamics of binding.

In previous studies, we often observed that the interaction of RPA with partner proteins has, in addition to a readily characterized interaction with RPA70N or RPA32C, a secondary interaction with the tandem high-affinity DNA binding domains RPA70AB (*e.g.* (13)). These interactions are invariably weaker than the contacts with the corresponding protein recruitment domain. An interaction of NEIL1 with RPA70AB would also support the previous report of NEIL1 interacting with the RPA70 subunit (12). To test this hypothesis, the ^15^N-^1^H HSQC spectrum of ^15^N-enriched RPA70AB was recorded with increasing amounts of NEIL1 (Fig. 2). This titration produced a gradual disappearance of RPA70AB signals due to line broadening. The titration was continued to an 8-fold excess of NEIL1, yet spectral changes continued to occur. The inability to saturate the effect indicates the interaction of NEIL1 with RPA70AB is weaker than the interaction with the RPA32C domain. Attempts to characterize the thermodynamics of binding by ITC revealed very small heats of binding and far from complete titration even at 2.5-fold molar excess, consistent with the weak binding affinity inferred from the NMR experiments.

### The high-affinity RPA binding motif is contained in the NEIL1 common interaction domain

**Figure 3:**
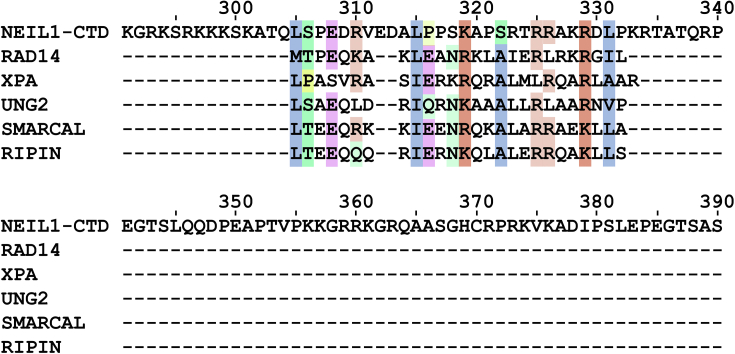
**Primary sequence alignment of NEIL1 CTD with known RPA32C binding motifs**. The NEIL1 C-terminal domain (residues 290–390) was aligned with known RPA32C binding motifs from various known RPA32C partner proteins Rad14, XPA, UNG2, SMARCAL1, and TIPIN (14, 16, 17, 52, 53). The degree of transparency of each color is proportional to the extent of conservation. Hydrophobic residues are colored in blue, positively charged residues in orange, negatively charged in *pink*, and polar residues in *green*.

All NEIL1 interactions with proteins characterized to date occur through the disordered C-terminal region (NEIL1_290-390_) termed the Common Interaction Domain (CID) (Fig. 1*A*). In order to locate more precisely the high-affinity RPA binding motif of NEIL1, a multiple sequence alignment was generated of NEIL1 CID using the RPA32C binding motifs identified for five other RPA binding partners (Fig. 3). The similarity of the sequences was sufficient to to identify a putative RPA-binding motif in the first half of the NEIL1 CID, within NEIL1 residues 305 to 331.

**Figure 4:**
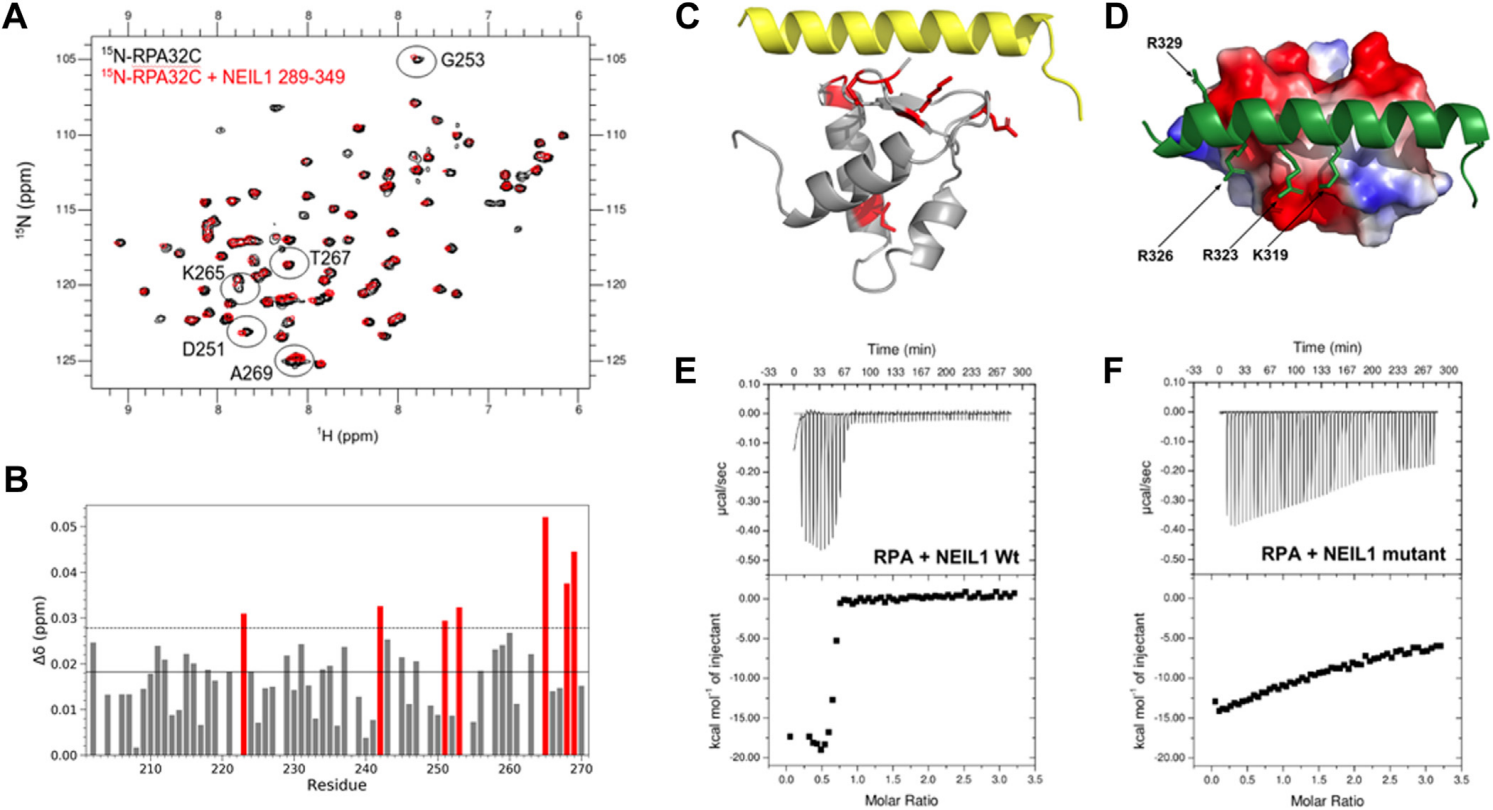
**Mapping and Disruption of NEIL1 Interaction with RPA32C.***A*, ^15^N-^1^H HSQC spectra were recorded at 800 MHz of ^15^N-enriched RPA32C in the absence (*black*) or presence (*red*) of 4 M equivalents of NEIL1_289-349_. Spectra were acquired at 25 °C in a buffer containing 25 mM Tris-HCl pH 7.5, 150 mM NaCl, and 1 mM DTT. The resonances with the largest CSPs are identified by circles and residue number. *B*, plot of NMR CSPs as a function of residue number. The mean of all CSPs is identified by the solid line and the standard deviation over the mean is identified by the thin dotted line. The most significantly perturbed residues are colored red. *C*, map of the CSPs on the homology model of the complex of RPA32C and the NEIL1_289-349_. The same significantly perturbed residues as in *B* are colored red in the model, showing they map primarily to the known RPA32C interaction interface. *D*, electrostatic field of RPA32C shown on the homology model of the complex with NEIL1_289-349_ (*green*). Surfaces are colored red for negative charge, white for neutral, and blue for positive charge. The basic side chains in NEIL1 that complement the acidic RPA32C surface and were mutated are identified with arrows. *E and F*, isothermal titration calorimetry of RPA titrated with NEIL1 wild-type (*E*) and mutant (*F*). The data were acquired at 25 °C in a buffer containing 50 mM HEPES (pH 7.5), 150 mM NaCl, 10% glycerol and 0.5 mM TCEP. The dissociation constant (Kd) for wild-type NEIL1 is 20 nM, which is consistent with a previously reported value (12). The binding of the mutant is so weak that the binding affinity cannot be determined by this approach.

To test this hypothesis, ^15^N-^1^H HSQC NMR spectra of ^15^N-enriched RPA32C were acquired in the absence or presence of a fragment of NEIL1 CID containing the putative RPA binding motif (NEIL1_289-349_). A comparison of the two spectra revealed significant CSPs throughout the RPA32C spectrum (Fig. 4, *A* and *B*), indicating that NEIL1_289-349_ does indeed contain the RPA interaction motif. Mapping of the CSPs onto the structure of RPA32C shows that the most strongly perturbed signals correspond to residues located on the same surface of RPA32C as observed for other RPA32C-binding proteins (*e.g.* XPA, UNG2, SMARCAL1, TIPIN (Fig. 4*C*), (14, 15, 16, 17). Based on these results, it was evident that a high-quality homology model of the complex of RPA32C and the NEIL1 RPA binding motif could be generated using the co-crystal structure of the complex of RPA32C with a peptide fragment of SMARCAL1 (PDB: 4MQV) as a template.

### Structure-based mutations of NEIL1 CID specifically disrupt the interaction

To test the functional relevance of the interaction of NEIL1 with RPA32C, we set out to design a NEIL1 variant that is defective in interactions with RPA32C. We began by using the homology model of the RPA32C-NEIL1_289-349_ complex to examine the interface (Fig. 4*C*). A combination of both electrostatic and hydrophobic interactions is found (Fig. 4*D*). The electrostatic component involves the acidic surface of RPA32C complemented by multiple basic residues at the surface of NEIL1. Since electrostatic interactions are long-range in nature and the interface between the two proteins covers a significant surface area, single-site mutations, even as charge reversals, are not likely to significantly decrease binding. Hence, a multi-site charge reversal mutation was designed, which we surmised would specifically and significantly inhibit the interaction between NEIL1 and RPA. Four mutations K319E, R323E, R326E and R329E were introduced into NEIL1, the protein was expressed, and an ITC titration was performed to compare the interaction of the wild-type and mutant NEIL1 proteins with RPA (Fig. 4, *E* and *F*). The Kd value of 20 nM for the wild-type protein is ∼10-fold lower than the value measured for RPA32C alone, consistent with RPA32C serving as the recruitment domain with only a minor contribution from the much more weakly binding RPA70AB. The ITC data show that the four mutations reduce the affinity so drastically it is not possible to extract a binding constant, indicating that interaction with RPA is indeed severely impeded.

### The RPA-interaction domain does not impact NEIL1 glycolytic activity

Initial characterization by FM-HCR revealed a counterintuitive increase in the repair of 8oxoG:C in NEIL1-depleted cells (Fig. S1*A*). This increase in repair efficiency was rescued by complementation by either WT or mutant NEIL1. Since 8oxoG:C is not among the preferred substrates for NEIL1, it is possible that NEIL1 binds non-productively and prevents other glycosylases from repairing the DNA lesion. Consistent with this model, when NEIL1 was depleted from OGG1 knockout cells, no change in repair efficiency was observed (Fig. S1*B*). Together, these findings are consistent with both WT and mutant NEIL1 having similar effects on BER in cells, but they do not directly assess the activity of the two variants. Furthermore, the OGG1 knockout cells were found to have been contaminated with *mycoplasma* after receipt in our laboratory, limiting our confidence in the data.

**Figure 5:**
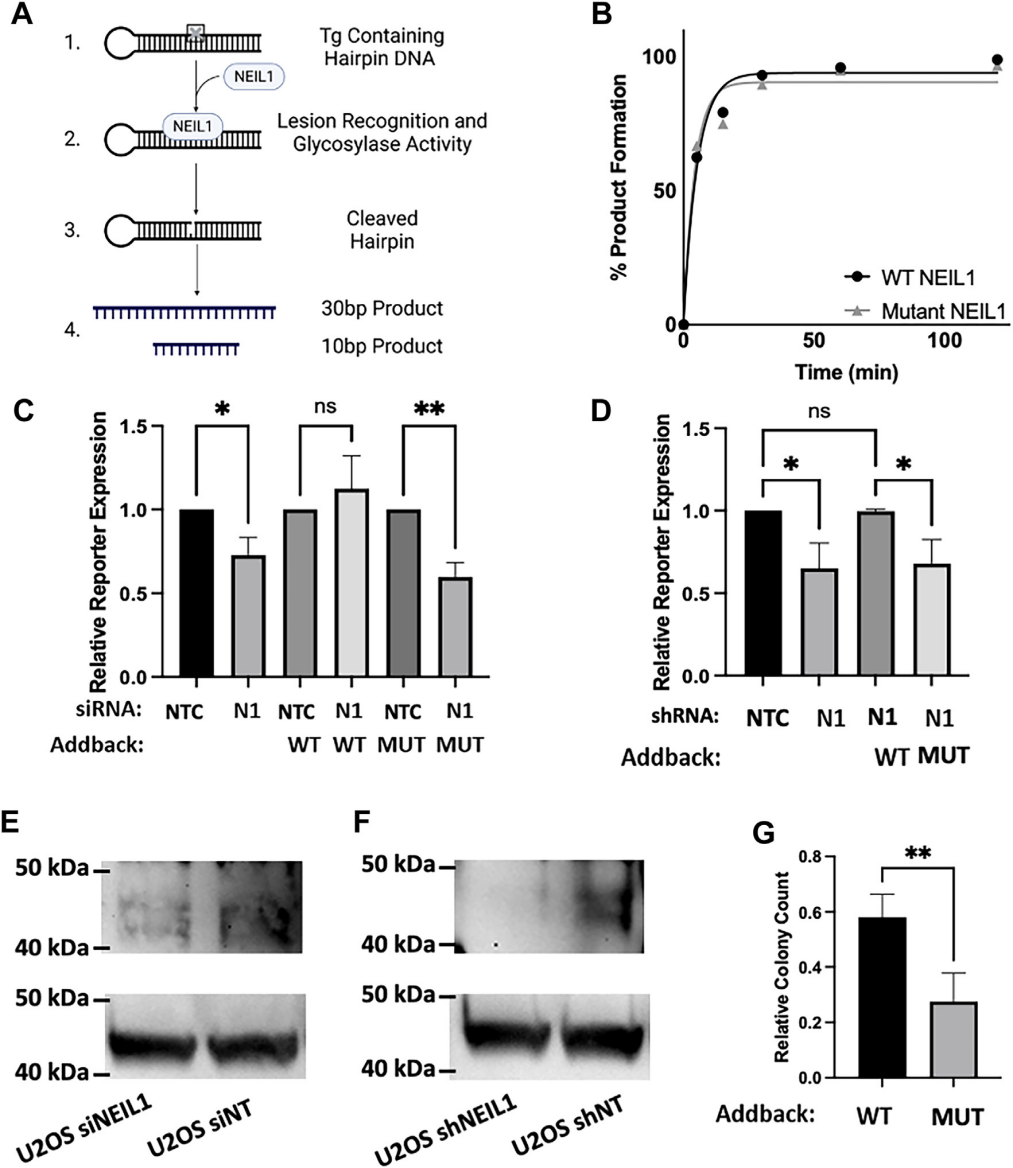
**DNA repair capacity and sensitivity to 4NQO.***A*, Hairpin-based biochemical assay for NEIL1 glycosylase activity. A 40 nt oligonucleotide containing a thymidine glycol lesion (*grey* ‘x’) (step 1) was incubated with WT or mutant NEIL1 for 24 h [steps 2–3]. Denaturing gel electrophoresis [step 4] allows for the resolution of repair products and image quantitation for determining reaction progress. *B*, time course showing the appearance of the cleaved product in both WT and mutant NEIL1 treated lesion-containing hairpin, quantified from the gels in Fig. S2. *C*, FM-HCR analysis of NER in U2OS cells complemented with wild-type (WT) or mutant (MUT) NEIL1 following treatment with non-targeting siRNA (NTC) or siRNA-mediated depletion of NEIL1 (N1). The abbreviations under each column in the plot indicate which siRNA and plasmid (if applicable) were transfected into the cells. For each addback condition, relative reporter expression was calculated by dividing fluorescent reporter expression in cells with siRNA-mediated depletion of NEIL1 by reporter expression in cells treated with non-targeting control. Western blot analysis of siRNA-mediated KD and addback of plasmid-encoded NEIL1 variants confirms expected changes in NEIL1 expression levels. Bar graphs show the mean ± SD, n = 3 with One-way ANOVA significance *p* ≤ 0.05. *D*, FM-HCR analysis of DNA NER capacity following shRNA-mediated knockdown of NEIL1 in U2OS cells. KD cells were complemented with plasmids encoding wild type or mutant NEIL1 as in panel *C*, bar graphs show the mean ± SD, n = 3 with One-way ANOVA significance *p* ≤ 0.05. *E*, Western blot of siRNA-mediated KD to validate expected changes NEIL1 protein levels with β-actin as a loading control. *F*, shRNA-mediated KD of NEIL1 was validated using Western blot analysis to confirm expected changes NEIL1 protein levels with β-Actin as a loading control. *G*, clonogenic survival assay of HAP1 NEIL1 knockout cells complemented with wild type or mutant NEIL1 and treated with 5 nM 4NQO. Relative colony count refers to the ratio of the number of colonies counted in the presence of 4NQO divided by the number of colonies counted in the vehicle control. Bar graphs show the mean ± SD, n = 3 with paired *t* test, significance is indicated using the following symbols: ns = not significant; ∗ = *p* ≤ 0.05, ∗∗ = *p* ≤ 0.005.

To investigate the impact of the mutations on NEIL1 activity directly, an *in vitro* biochemical assay using either the purified WT or RBD mutant NEIL1 protein incubated with a hairpin containing a thymidine glycol lesion was used to measure the rate at which the WT and RBD mutant cleave oxidative lesions (Fig. 5*A*). Gel-based analysis of the repair products (Fig. S2) and quantitation by densitometry (Fig. 5*B*) indicated that both enzymes cleave the thymidine glycol lesion at similar rates (comparison of best fits test, *p* = 0.950, F = 0.111). These data confirm that mutation of the RPA-binding domain does not have a detectable effect on the glycolytic activity of the enzyme *in vitro*. Additionally, incubation of undamaged hairpin DNA or a hairpin containing a uracil at the same position with NEIL1 enzymes confirmed the specificity of the assay (Fig. S2).

### Loss of NEIL1-RPA interaction results in a mild nucleotide excision repair defect

To assess the functional implications of the RPA-NEIL1 interaction, targeted knockdown of NEIL1 followed by rescue with either WT or mutant NEIL1 was carried out in U2OS, and cells were analyzed for DNA repair capacity by FM-HCR analysis. 72 h after U2OS cells were transfected with the non-targeting siRNA (NTC) or NEIL1-targeting siRNA (N1), they were co-transfected with FM-HCR reporter plasmids and either pMax_WT_NEIL1 or pMax_Mutant_NEIL1. Unexpectedly, we observed a mild defect in the NER pathway. Comparison of NEIL1-depleted cells complemented with pMax_WT_NEIL1 (fourth column in Fig. 5*C*) or pMax_Mutant_NEIL1 (sixth column in Fig. 5*C*) showed that only WT NEIL1 rescues the NER defect observed in cells without complementation (second column in Fig. 5*C*). Similar results were obtained using constitutive knockdown of NEIL1 using shRNA in U2OS cells (Fig. 5*D*). NEIL1 depletion again resulted in a decreased in NER activity that was rescued by WT, but not mutant NEIL1. Transient and constitutive knockdowns of NEIL1 were confirmed at the protein level by Western blot (Fig. 5, *E* and *F*). β-Actin was included in each blot as a loading control. RT-qPCR analysis of transcript levels further corroborated the siRNA-mediated knockdown, though sample variability made this analysis less robust than the western analysis (Fig. S3). This method indicated an approximately 2.5 × 10^4^-fold increase in NEIL1 expression at the transcript level for both the mutant and WT addback (Fig. S3), confirming the overexpression detected by Western blot.

To reproduce these observations in a model that does not rely on RNAi-mediated depletion of NEIL1, we performed analogous experiments in HAP1 cells in which NEIL1 has been deleted using CRISPR-Cas9. The NEIL1 knockout cells were transfected using either pMax_EV, pMax_WT_NEIL1 or pMax_Mutant_NEIL1. The knockout and addbacks were validated at the protein level by Western blot as shown in Fig. S3. Consistent with the results in U2OS, complementation with the WT NEIL1 that is able to bind RPA results in a slight trend towards increased in NER capacity, whereas complementation with the empty vector or the RPA-binding-deficient NEIL1 mutant did not (Fig. S3). The effect of NEIL1 addback did not reach statistical significance in this cell line (*p* = 0.0781), however the overall trend matched our observations in other cell lines. Finally, to confirm that the modest changes in NER observed using plasmid-based assays are biologically relevant, we used a clonogenic survival assay following exposure of cells to 4NQO. This agent induces bulky mono adducts that are only known to be repaired by the NER pathway, and it is substantially more toxic to NER-deficient cells than it is to NER-proficient cells (18). Whereas NEIL1 expression levels had no effect on plating efficiency (Fig. S4), NEIL1 knockout cells complemented with pMax_WT_NEIL1 were significantly more resistant to 4NQO than NEIL1 knockout cells complemented with pMax_Mutant_NEIL1. By contrast, the sensitivity of WT control cells was unaffected by transfection with either pMax_WT_NEIL1 or pMax_Mutant_NEIL1 (Fig. 5*G*), indicating that overexpression of NEIL1 does not further enhance resistance to 4NQO in cells expressing basal levels of NEIL1. These data suggest that the NEIL1-RPA interaction protects cells from killing by 4NQO by promoting NER of the bulky DNA adducts produced in genomic DNA.

## Discussion

Since the discovery that NEIL1 is up-regulated in the S phase and is also able to process ssDNA (10, 19, 20), there has been a great deal of interest in understanding its role in replication-associated DNA repair (11, 16, 21, 22). As the BER pathway requires the generation of potentially toxic DNA structures in the context of replication, such as abasic sites and strand breaks, it is essential that these two cellular processes are regulated and coordinated. The coordination between the two DNA processing pathways relies on a network of key protein-protein interactions, such as that between RPA and NEIL1 as characterized here. Understanding how these interactions occur at the molecular level provides valuable insights for testing functional models and elucidating the mechanism of the interplay between replication and BER.

In this study, we have shown that unlike inferences drawn in a previous study (12), the primary contact between NEIL1 and RPA is the RPA32C protein recruitment domain. We have also confirmed the existence of the interaction with the RPA70 subunit as reported previously (17), which we mapped to the tandem RPA70AB domains and showed by ITC that it is considerably weaker than the interaction with RPA32C. A homology model of the complex of RPA32C and the NEIL1 RPA binding motif in the CID was then used to design specific mutations at the NEIL1-RPA interface to inhibit the interaction. We went on to show that a NEIL1 variant with four charge-reversal mutations effectively suppresses the physical interaction with RPA. This NEIL1 mutant serves as a valuable reagent for investigating the functional significance of the NEIL1 interaction with RPA and its effect on the coupling of replication and BER, both *in vitro* and in cells.

The relevance of the interaction between RPA and NEIL1 is underscored by the recent discovery that in mitochondria the functional homolog of RPA, mtSSB, also interacts with NEIL1 in the CID region of the NEIL1, albeit in a different motif from that used by RPA (23). To our knowledge, mtSSB has no known functions outside of mitochondria, so we view the interaction *via* the CID as functionally homologous with respect to the primary BER function of NEIL1. At present, there are no reports of any role for mtSSB in NER in mitochondria.

Here we have shown that the molecular basis of the interaction between NEIL1 and RPA32C is very similar to the previously characterized interaction of another DNA glycosylase involved in the initiation of the BER pathway, UNG2 (17). Like the NEIL1 CID, UNG2 has an RPA32C binding motif in a disordered domain adjacent to the catalytic domain, implying there may be a similar mechanism of binding. A recent study using IPOND (isolation of protein on nascent DNA) revealed that NEIL1, UNG2, and other glycosylases are found at replication forks (22). Different glycosylases function to repair different types of DNA base damage. Hence, the ready availability of glycosylases at the replication fork and the need to rapidly repair damaged bases without accumulating repair intermediates that are more toxic than the initial base lesions may be regulated by their interaction with RPA. RPA may stimulate pre- and post-replicative BER in dsDNA flanking ssDNA but inhibit BER in ssDNA, preventing the formation of toxic strand breaks.

Finally, it appears that although the interaction between NEIL1 and RPA is dispensable for the activity of NEIL1 against oxidative lesions in duplex DNA, the interaction plays an unexpected role in cellular NER capacity. Knockdown of NEIL1 using either siRNA or shRNA results in a mild NER defect that is only rescued when NEIL capable of binding RPA is re-introduced to these cells. Though the knockdown of NEIL1 was relatively inefficient in our hands, this finding is further supported through the introduction of these same proteins into NEIL1 knockout cells using both FM-HCR and a clonogenic survival assay measuring sensitivity to 4NQO. This agent was chosen because it induces bulky DNA mono adducts that are only known to be repaired by the NER pathway. By contrast, other agents like cisplatin also induce inter-strand crosslinks (24), and lesions such as those generated by aflatoxin are substrates for NEIL1 (25). Like many agents that induce bulky DNA adducts, 4NQO induces some oxidative DNA lesions (26) but these are unlikely to explain the differential sensitivity of cells expressing the mutant NEIL1 because it appears to be fully active towards oxidative DNA lesions (Fig. 5*B*). Collectively, the data are consistent with the rescue of NER proficiency only when NEIL1 is capable of binding of RPA.

Our results add to previous work indicating crosstalk between DNA glycosylases and NER (27, 28, 29, 30, 31, 32). Noting that NEIL1 is capable of binding and excising some types of bulky DNA adducts (25, 29), our data may suggest a possible handoff mechanism wherein NEIL1 recruits XPA to sites of DNA damage *via* shared interactions with RPA. However, since NEIL1 also interacts with numerous other proteins (33, 34, 35), it is possible that the four charge reversal mutations we report here disrupt additional NEIL1 protein interactions that might be required for efficient NER. Additional work is needed to determine why partial NEIL1 depletion is sufficient to cause a defect in NER and to verify our model in which NEIL1 interferes with OGG1-dependent repair of 8oxoG:C repair. In conclusion, our findings provide new insights into the role of interactions between RPA and NEIL1 in multiple DNA repair pathways and suggest previously unrecognized targets for therapeutic inhibition of DNA repair in cancers.

## Experimental procedures

### Plasmids

The cloning of RPA constructs is described elsewhere (14, 36, 37, 38). NEIL1 expression vectors were a kind gift from Drs. Muralidnar Hedge and Sankar Mitra. The NEIL1 K319E/R323E/R326E/R329E mutant was generated using the Quick Change protocol as previously described (39). PCR primers were 5′-CGAGACACGAGAGGCAAAGGAAGACCTTCCTAAGAGGAC-3′ and 5′-CCTCTCGTGTCTCGGAAGGGGCCTCGCTTGGA-3′. Correct incorporation of the mutations was confirmed by DNA sequencing (Genewiz). The WT and mutant genes were then amplified from these expression plasmids using the PCR primers:

5′-GCGCTAGCAGCACCCATATGCCTGAG-3′

5′-ACGAAGCTTTCAGTGGTGGTGGTGGT-3′

These were subcloned into the pMaxCloning vector (Lonza, VDC-1040) *via* NheI and BamHI sites to generate the pMax_WT_NEIL1 and pMax_Mutant_NEIL1 expression plasmids that were utilized throughout this analysis.

### Expression, purification of NEIL1 and RPA

RPA70N, RPA32C, RPA70AB, and full-length RPA were expressed and purified as previously described (14, 37, 40, 41). Wild-type and mutant NEIL1 proteins were expressed from a pET22b vector harboring the wild-type or mutant NEIL1 gene fused with C-terminal 6-His tag transformed into Rosetta 2 (DE3) *E. coli* cell line. A fresh colony was used to inoculate a starter culture of 100 ml of autoclaved Terrific Broth (TB) medium supplemented with ampicillin in 250 ml baffled flask. After overnight growth at 37 °C with shaking at 230 rpm, 15 ml of starter culture was used to inoculate each 1L of TB culture medium supplemented with ampicillin in a 2L baffled flask. Growth was carried out at 37 °C with shaking at 230 rpm until OD_600_ reached 0.8. Temperature was then decreased to 18 °C and NEIL1 protein expression was induced by addition of 0.5 mM of isopropyl-D-1-thiogalactopyranoside (IPTG). After overnight growth, cells were harvested by centrifugation (6000 rpm, 4 °C, 20 min). The supernatant was discarded and cell pellets were stored at −20 °C until purified.

To purify NEIL1, cell pellets were resuspended in Lysis buffer (20 mM Tris-HCl pH 7.5, 500 mM NaCl, 5% w/v glycerol, 20 mM imidazole, 1% w/v NP-40, 0.1 mM PMSF, 5 mM beta mercapto-ethanol, protease inhibitor tab) using 5 ml of lysis buffer per gram of cell pellet. The cell suspension was homogenized and lysis was carried out by sonication at 4 °C (10 min, 5 s “on”, 10 s “off”, 50% power). The lysate was centrifuged at 50,000*g* for 45 min at 4 °C. The supernatant was collected and filtered at 0.45 um. This lysate was applied to a nickel affinity column (HisTrap FF 5ml ©Ge Healthcare) pre-equilibrated with Ni-A buffer (20 mM Tris-HCl pH 7.5, 500 mM NaCl, 5% w/v glycerol, 20 mM Imidazole, 0.1 mM PMSF, 5 mM beta mercapto-ethanol). The resin was then washed with 20 CV of Ni-A buffer and the protein was eluted with 10 CV of Ni-B buffer (Ni-A + 300 mM imidazole). Elution fractions were analyzed by SDS-PAGE and fractions containing NEIL1 protein were pooled together and diluted 2.5-fold with dilution buffer (20 mM HEPES pH 7.5, 5% w/v Glycerol, 1 mM EDTA, 0.1 mM PMSF, 2.5 mM beta mercapto-ethanol). The diluted protein solution was loaded onto a tandem of pre-equilibrated Q and S columns in buffer A (20 mM HEPES pH 7.5, 125 mM NaCl, 5% w/v glycerol, 1 mM EDTA, 0.1 mM PMSF, 2.5 mM beta mercapto-ethanol). At this ionic strength, the Q column filtered out binding contaminants while NEIL1 flows through the resin and then binds to the S column. Subsequently, a 20 CV wash step with buffer A was applied and the Q column was removed. The protein was eluted using a linear sodium chloride gradient from 200 mM to 1 M over 10 CV. Fractions were analyzed by SDS-PAGE, and those that contained pure NEIL1 were pooled together and concentrated using an Amicon © cutoff 10 kDa. The concentrated protein was applied to a S75 SEC column pre-equilibrated with S75 buffer (20 mM HEPES pH 7.5, 200 mM NaCl, 5% w/v glycerol, 1 mM EDTA, 0.1 mM PMSF, 2.5 mM Beta mercapto-ethanol). Fractions containing the final pure protein, as confirmed by SDS-PAGE, were pooled together, aliquoted, flash-frozen in liquid nitrogen, and stored at −80 °C.

### Nuclear magnetic resonance

All NMR experiments were recorded at 25 °C on Bruker Avance III NMR spectrometers operating at 600 or 800 MHz in a buffer containing 20 mM HEPES pH7.5, 200 mM NaCl, 5 mM DTT, and 5% D_2_O. Data were acquired from 200 μl samples in 3 mm NMR tubes. ^15^N-^1^H SOFAST HMQC spectra were recorded using 32 scans, 1024 points in the direct ^1^H dimension and 128 points in the indirect ^15^N dimension, with a recycle delay of 200 ms. All data were processed using Topspin© (Bruker) and analyzed using CCPNMR analysis software (42). Chemical Shift Perturbations (CSP) were calculated using:

$$CSP=\left[\delta_{H}^{2}+\left(0.15\times \delta_{N}^{2}\right)\right]$$

CSPs were plotted using pyplot.

### Multiple sequence alignment

Protein sequences were obtained from the uniprotKB portal (https://www.uniprot.org) (43) and aligned using Clustal OMEGA (https://www.ebi.ac.uk/Tools/msa/clustalo/) (44). The alignment figure was generated using JALVIEW (45).

### Homology modelling of RPA32C in complex with NEIL1 RBS

The complex of RPA32C and the NEIL1 RPA Binding Motif (NEIL1 RBF) was generated using Modeller version 9.2 (46). The crystal structure of RPA32C with SMARCAL1 (PDB: 4MQV) was used as a template. 100 models were generated and the model with the lowest DOPE score was kept as the final model. The model is available in ModelArchive at https://www.modelarchive.org/doi/10.5452/ma-p9ihk.

### Isothermal titration calorimetry

Protein samples were dialyzed against the same buffer solution containing 50 mM HEPES (pH 7.5), 150 mM NaCl, 10% glycerol, and 0.5 mM TCEP. ITC data were collected using a Microcal VP isothermal titration calorimeter operating at 25 °C. Various concentrations of protein in the cell and the syringe were tested and the optimal concentrations from which the final parameters are reported were 50 uM in the cell and 150 uM in the syringe with an injection volume was 10 uL. The binding parameters for titrations that could be quantified are reported as the mean and standard deviation of three independent replicates. Data were analyzed using NITPIC software, version 1.2.7 (47).

### *In vitro* biochemical assay for NEIL1 activity

Three 40 nt oligonucleotides were obtained from commercial sources. Each oligonucleotide had the following sequence: (5′ATCTATCCGAXCACGCACCGACCCTTCCCTCGGTGCGTGGTCGGATAGAT3′), where ‘X’ was a thymine glycol, uracil, or cytosine. The thymine glycol-containing hairpin was used to measure NEIL1 activity. The oligonucleotides were designed such that they form a hairpin in which the position of interest pairs with a guanine base. The uracil-containing hairpin was used with a commercially available uracil glycosylase as a positive control for the assay principle. The cytosine-containing hairpin served as a damage-free negative control that would not be processed by either a uracil glycosylase or NEIL1. To form hairpins, 0.1 nmol of each oligonucleotide was diluted to 4 nM and subjected to heating at 90 °C for 5 min followed by cooling to 25 °C at a rate of 0.5 °C per second in a Pro-Flex thermocycler (ThermoFisher, 4484073). Thymidine glycol or control hairpins were incubated with 1.0 μM of purified recombinant WT or mutant NEIL1 or no enzyme (negative control) in the presence of 50 mM HEPES, 150 mM NaCl, 5% glycerol, and 4 μM BSA. The concentration of purified proteins was confirmed using a BCA assay kit (ThermoFisher, 23225). Conditions for this assay were based on those previously reported (48). As a positive control, the uracil-containing hairpin was incubated using the USER II enzyme under conditions recommended by the manufacturer, New England Biolabs. Reactions were incubated at 37 °C for 0 min, 5 min, 15 min, 30 min, 1 h, 2 h, and 24 h with reactions stopped using the formamide containing ThermoScientific Gel Loading Buffer II (AM8546G) and heating for 5 min at 95 °C, followed by storage at 4 °C. Reaction products were analyzed using a 20% non-denaturing polyacrylamide gel for 1 h at 125 V, stained with 1× SybrGold (ThermoScientific, #38-0221-01) for 30 min and imaged using the iBright Imaging System. The smaller single-stranded 10 bp fragment product was not visible, likely reflecting the smaller amount of DNA in the band and weaker staining of ssDNA. An unexpected band that migrated slightly ahead of the substrate band at 40 nt appears to be a conformational isomer of the hairpin-forming oligonucleotide that is refractory to NEIL1 cleavage. Gel quantitation was performed using GelAnalyzer software. Densitometry was carried out to estimate the intensity of the visible 30 bp product and 40 bp substrate bands after background subtraction. The band intensity was then corrected to reflect the size of the DNA fragment. The corrected optical density value for the product band was then divided by the sum of the density values for the product and substrate bands and converted to a percentage to estimate reaction progress.

### Knockdown of NEIL1 using siRNA

U2OS (ATCC, HTB-96) cells were transfected with an siRNA pool targeting NEIL1 (Dharmacon, L-008327-00-0005) or a non-targeting control siRNA (Dharmacon, D-001810-01-20) as a control. Using a 10 μl tip, 200,000 cells were transfected with 100 pmol of siRNA using a Neon Transfection system (ThermoScientific, MPK5000) at 1230 V, 10 ms, and 4 pulses. Knockdown was confirmed by Western blot and determined to reach maximal efficiency at 96 h. These conditions were selected for downstream experiments.

### Western blot analysis of NEIL1 variants

Approximately 2.5 x 10^6^ cells were collected and lysed using NETN 150 mM NaCl, 1 mM EDTA, 50 mM Tris-HCl pH 7.5, 1% NP40) buffer. Protein concentration in the lysate was determined using a BCA assay assay kit (ThermoFisher, 23225). 50 μg of whole cell extract was added per well of a 15% polyacrylamide gel and run at 200 V for 2.5 h. Proteins were then transferred to a PVDF membrane *via* wet transfer at 100 V for 1 h. This membrane was then blocked using 5% dry milk in PBS + 0.5% Tween-20 for 1 h followed by three PBST washes. Primary antibody incubation used either a 1:500 dilution of rabbit anti-human NEIL1 polyclonal antibody (Proteintech, 12145-1-AP) or a 1:200 dilution of mouse anti-human β-Actin monoclonal antibody (Santa Cruz, C4; sc-47778) overnight in PBST + 5% dry milk. The blot was then washed three times using PBST followed by secondary incubation using a 1:10,000 dilution of goat anti-rabbit H + L antibody (Bio-Rad, #1706515) in PBST + 5% dry milk for 1 h. Finally, the blot was imaged using an iBright 1500 imaging system after three PBST washes.

### Constitutive knockdown of NEIL1 using shRNA

U2OS cells were transduced using MISSION shRNA lentiviral particles containing either non-targeting shRNA (Millipore-Sigma, SHC016V) or NEIL1-targeting shRNA (Millipore-Sigma, TRCN0000049708). The NEIL1-targeting shRNA containing lentiviral particles expressed an interfering RNA that targets the sequence GCTACGAAACCTAGCGGATAA. Cells were plated at 1 × 10^5^ cells per well in 2 ml DMEM + 10 FBS in 6 well plates and allowed to seat for 24 h. Hexadimethrine bromide was added at 8 μg/ml followed by the introduction of viral particles at an MOI of 1.0. Particle-containing media was removed and replaced 24 h later with fresh media. At the 72 h time point, fresh media containing 1.0 mg/ml puromycin was added to each well. After 2 days, the cells were transferred to a T75 flask with puromycin-containing media. Puromycin selection was allowed to continue for 1 week. Cells were cultured in the absence of selection for 48 h prior to validation by Western blot and used in experiments.

### FM-HCR analysis of DNA repair capacity in cells complemented with NEIL1

72 h after siRNA transfection, cells were transfected with 750 ng of either pMax_WT_NEIL1 or pMax_Mutant_NEIL1 using Lipofectamine3000 (ThermoScientific, L3000001). After an additional 24 h in culture, the cells were then trypsinized, collected by centrifugation, and transfected with Fluorescence Multiplex-Host Cell Reactivation (FM-HCR) reporter plasmids using the Gene Pulser MXCell Plate Electroporation System (Bio-Rad, #165-2670) using conditions 260 V, 950 μF. FM-HCR assays were carried out as previously described using reporter plasmids expressing green fluorescent protein (GFP), orange fluorescent protein (mOrange), and mPlum red fluorescent protein (mPlum) (49). Reporter plasmids were prepared as a cocktail containing pmax_GFP plasmid damaged with 800 J/cm^2^ UVC radiation (herein referred to as GFP_UV) and an undamaged pMax_mPlum control as well as an undamaged cocktail containing pMax_GFP and pMax_mPlum. To analyze the repair of 8oxoG:C, cells were transfected with a cocktail including undamaged pMax_mPlum plus mOrange_8oxoG, or an undamaged cocktail containing pMax_mPlum and pMax_mOrange. The resulting fluorescence was then measured using an Attune N x T Flow Cytometer (ThermoScientific). NER capacity was also determined in HAP1 NEIL1 knockout cells obtained from (Horizon Discovery). These cells were transfected with 750 ng of an empty pMax vector (pMax_EV), pMax_WT_NEIL1, or pMax_Mutant_NEIL1 using Lipofectamine3000 and analyzed *via* FM-HCR as previously described (49). This analysis was completed in an identical manner using the constitutive shRNA-based KD of NEIL1 and isogenic non-targeting control cells produced in the U2OS background. For these experiments, both NEIL1 KD and control U2OS cells were transfected with the aforementioned cocktail as well as 750 ng of either pMax_EV, pMax_WT_NEIL1, or pMax_Mutant_NEIL1 using Lipofectamine 3000 24 h after cell plating. After another 24 h, cells were collected *via* trypsinization and FM-HCR cocktails were transfected *via* electroporation using the MXCell System. Cells were then collected and analyzed *via* flow cytometry as described previously (50).

### Clonogenic survival assay

NEIL1 knockout cells were transfected with 750 ng pMax_EV, pMax_WT_NEIL1, or pMax_Mutant_NEIL1 using Lipofectamine3000 and allowed to incubate for 24 h. Cells were then collected and re-plated at 500 cells/well with either 5 μM 4-nitroquinolone *N*-oxide (4NQO) (Sigma, N8141) or a dimethyl sulfoxide (DMSO) (Sigma, D8418) control. The cells were left for 7 days and then fixed using 100% methanol (MeOH) (VWR, BDH2029) and stained with a crystal violet (Sigma, C6158) solution (20% MeOH, 80% H_2_O, 0.05% crystal violet). Visible colonies containing 50 or more cells were counted and recorded.

### Cell lines

U2OS cells were obtained from ATCC (HTB-96). The OGG1−/− cell line was a gift from Patricia Opresko and has been reported previously, see clone 13 in (51). Stocks containing approximately 2 to 4∗10^6^ cells were seeded into T25 flasks with 5 ml DMEM + 10% FBS. After the first passage, stocks were made to ensure cells were used with a similar low passage number for all experiments. HAP1 WT and NEIL1 knockout cells were purchased from Dharmacon and also received as frozen stocks of approximately 1 to 3∗10^6^ cells. These cells were thawed and seeded into T25 flasks in 5 ml of IMDM + 10% FBS media. These cells were also periodically stocked and frozen to ensure the maintenance of cells at their lowest possible passage. All cells were routinely checked for *mycoplasma* contamination using either the ATCC Universal *Mycoplasma* Detection Kit (30-1012K) or the Lonza MycoAlert *Mycoplasma* Detection Kit (75860-358). Cell counts and viability were determined using a ViCELL Cell Viability Analyzer.

## Data availability

All data are contained within the manuscript. All reagents are available upon request from the authors.

## Supporting information

This article contains supporting information.

## Conflict of interest

The authors declare that they have no conflicts of interest with the contents of this article.
