# Supplementary material for: Molecular basis and functional consequences of the interaction between the base excision repair DNA glycosylase NEIL1 and RPA
Source: J Biol Chem. 2024 Jul 25;300(9):107579. doi: 10.1016/j.jbc.2024.107579 (PMC11387677; doi:10.1016/j.jbc.2024.107579)
Supplement: Supplementary Figures [file mmc1.pdf]

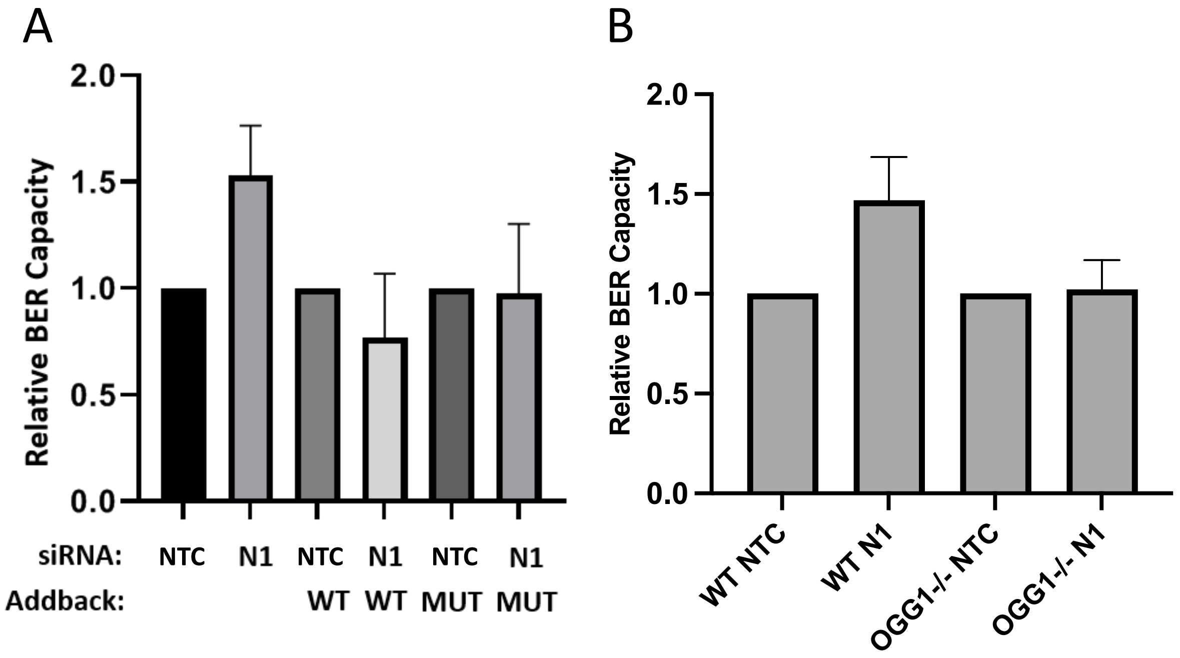

Supplementary Figure 1. FM-HCR analysis of 8oxoG:C repair in NEIL1 deficient cells. A. FM-HCR analysis of 8oxoG:C base excision in U2OS cells complemented with wild type (WT) or mutant (MUT) NEIL1 following treatment with an non-targeting control siRNA (NTC) or siRNA-mediated depletion of NEIL1 (N1). B. FM-HCR analysis of 8oxoG:C base excision in wild type (WT) U2OS cells or OGG1 knockout cells (OGG1-/-). -/-). Bar graphs show the mean +/- SD, n=3. Note: the cell lines used in panel B tested positive for mycoplasma after receipt in the Nagel lab.

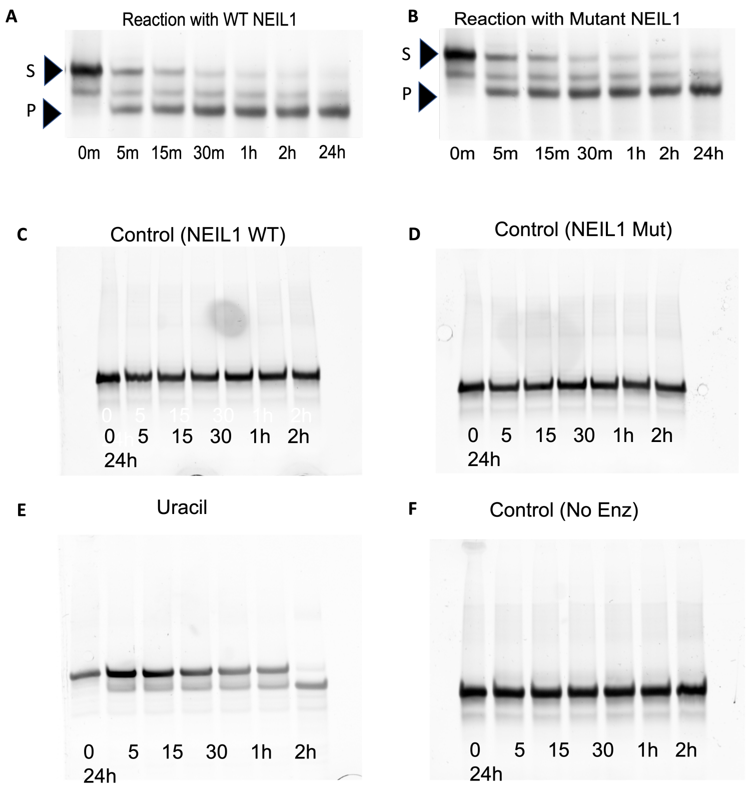

Supplementary Figure 2. Glycosylase Hairpin Activity Assay A. Processing of a thymidine glycol containing oligonucleotide by WT NEIL1 over a 24h period. Appearance of the bottom-most product band (P) and disappearance of the top-most substrate band (S) shows complete cleavage of the hairpin. **B.** An identical analysis was carried out using mutant NEIL1. **C.** Wild type NEIL1 was incubated for 24h with a lesion-free hairpin forming oligonucleotide as a control **D.** The lesion-free hairpin-forming oligonucleotide was incubated with mutant NEIL1 as a negative control. **E.** An oligonucleotide containing a uracil lesion was incubated with the USERII enzyme (NEB) for 24 hours as a positive control to validate the excision reaction. **F.** A second

negative control was performed using the thymidine glycol containing lesion with no enzyme included to confirm that no excision occurs without a glycosylase present.

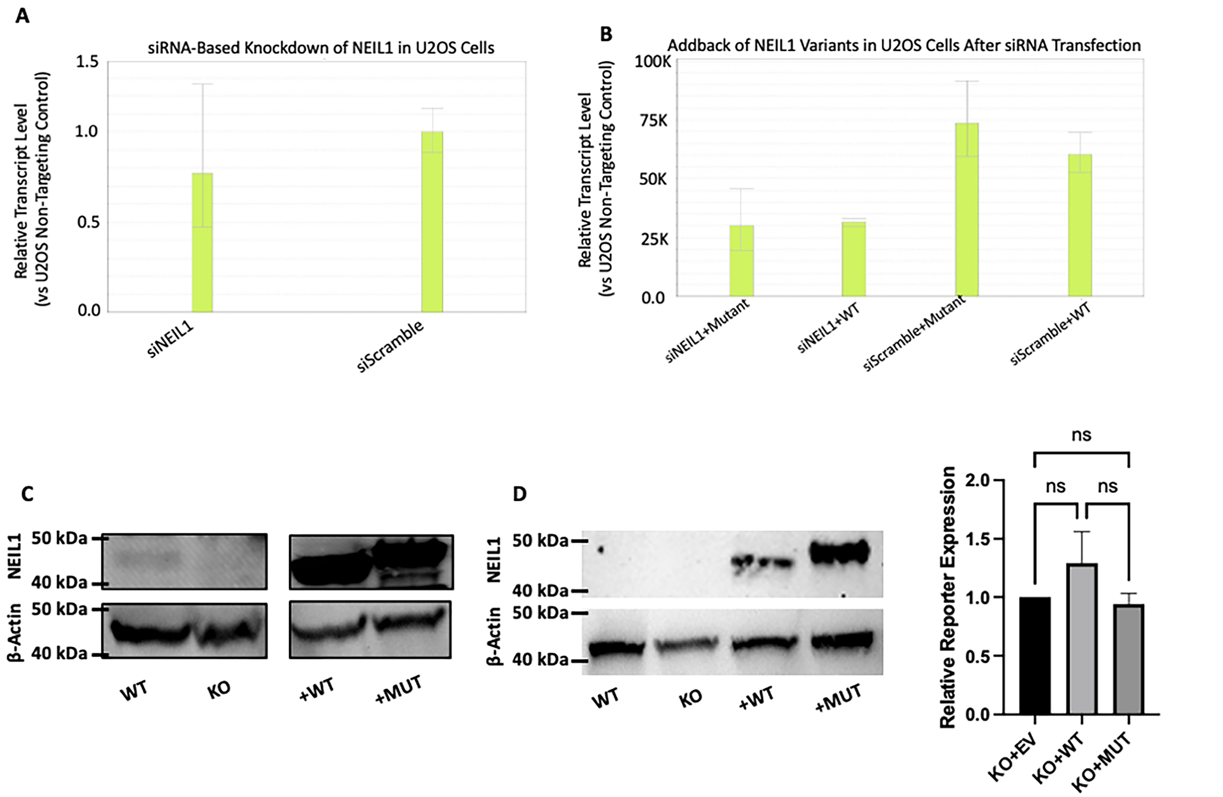

Supplementary Figure 3. Validation of U2OS siRNA and HAP1 knockout experiments A. RT-qPCR analysis of NEIL1 siRNA-mediated KD in U2OS cells compared between siNEIL1 and non-targeting control in U2OS. **B.** RT-qPCR analysis confirming NEIL1 addback in siRNA transfected samples. Wild type and mutant addback of NEIL1 in both non-targeting control and siNEIL1 transfected U2OS cells. **C.** Western blot validation of HAP1 WT (**WT**) and NEIL1 knockout cells with wild type and mutant addback in NEIL1 knockout cells. **D.** Western blot with lower exposure confirming over-expression of both wild type and mutant protein after addback in NEIL1 knockout cells. **E.** FM-HCR analysis of NER capacity in HAP1 NEIL1 knockout cells transfected with an empty vector (EV) control or vectors expressing wild type (WT) or RPA binding mutant (MUT) NEIL1. Bar graphs show the mean +/- SD, n=3 with one-way ANOVA, significance $p \leq 0.05$.

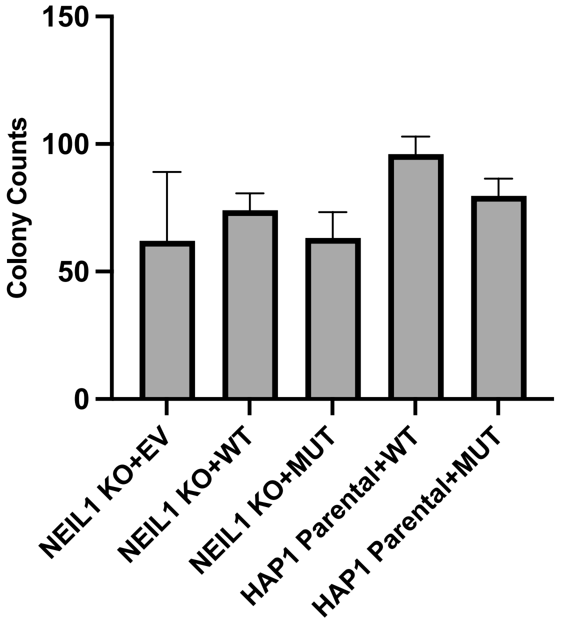

Supplementary Figure 4. Raw colony counts for cells expressing WT or mutant NEIL1. NEIL1 KO cells and parental controls formed colonies at similar rates, and overexpression of NEIL1 did not affect the ability of cells to form colonies. Cells were transfected with an empty vector (+EV), wild type NEIL1 (+WT), or mutant NEIL1 (+MUT). Bar graphs show the mean +/- SD for 3 or more independent measurements.
